# Properties of polyetheretheretherketone (PEEK) implant abutments: A systematic review

**DOI:** 10.4317/jced.59466

**Published:** 2022-04-01

**Authors:** Romaisa Ghazal-Maghras, Jaime Vilaplana-Vivo, Fabio Camacho-Alonso, Yolanda Martínez-Beneyto

**Affiliations:** 1 DDS. Faculty of Medicine-Dentistry. University of Murcia, Spain; 2 DDS, PhD, Assistant Professor. Department of Surgery. Faculty of Medicine-Dentistry. University of Murcia, Spain; 3 DDS, PhD. Professor. Department of Dental Surgery. Faculty of Medicine-Dentistry. University of Murcia, Spain; 4 DDS, PhD. Doctor Contracted Professor. Department of Oral Surgery. Faculty of Medicine-Dentistry. University of Murcia. Institute of Biosanitary Research (IMIB), Spain

## Abstract

**Background:**

The main objective of the present systematic review is to know the mechanical and functional properties of PEEK (polyetheretheretherketone) abutment and to find out if it is a potential substitute for titanium abutments.

**Material and Methods:**

An electronic search was conducted in 5 databases: Medline (Pubmed), SciELO, Cochrane, Web of Science (WOS) and Google Scholar. Studies published from 2018 to 2020 and written in English were included. The protocol of this systematic review was registered in PROSPERO (ID 274834). Subsequently, data extraction and quality analysis were performed according to the modified CONSORT guidelines.

**Results:**

Initially, a total of 976 articles were obtained. Using Mendeley Desktop, duplicates were discarded, reducing the number of articles to 483. After reading abstracts, 448 articles were excluded. Finally, 35 full-text articles were analysed, of which 5 articles were included in this systematic review.

**Conclusions:**

The available evidence shows that PEEK implant abutments do not have sufficient biomechanical requirements to replace the definitive titanium abutment. However, it is considered as an alternative and provisional material, especially if placed in the anterior region.

** Key words:**Abutment, polyetheretheretherketone, titanium, dynamic fatigue, fracture toughness, loss of torque.

## Introduction

Pure titanium (Ti) is the first material used commercially for dental implants, (1). This material and its alloys generally possess high corrosion resistance, good biocompatibility, good osseointegration and excellent mechanical properties, leading it to be considered the gold standard for dental implants and abutments (2,3).

In recent years, Polyetheretheretherketone (PEEK) is being considered as an alternative to titanium and ceramic (4). In the late 1970s, a high-performance thermoplastic polymer was introduced for the first time to replace metal implants and abutments, called polyetheretheretherketone, commonly known as PEEK. (3) Polyetheretheretheretherketone (PEEK) and polyetheretherketone ketone (PEKK) are two groups belonging to the Polyaryl-etherketone family. PEEK has a straight chain structure, consisting of an aromatic ring with combinations of ketone (-CO-) and ether (-O-) functional groups between the aryl rings (4-8). It can be manufactured by CAD-CAM or compression moulding (7,9). It was initially used in orthopaedic applications (spinal surgery, fracture fixation devices, joint replacement and maxillofacial surgery) (10). In April 1998 it was commercialised for the first time by the British company Invibio as an implant material for use in dentistry (11).

PEEK has an aesthetic tooth-like whitish colour, is biocompatible and fracture resistant (3,12). In addition, it has excellent mechanical and chemical properties even at high temperatures (13,14). It has a glass transition temperature of around 143°C and melts at approximately 343°C (7,8). It is very resistant to corrosion (7,8). It is highly resistant to thermal, aqueous and chemical degradation except for 98% sulphuric acid (10,15). Its modulus of elasticity is 3.6 GPa and 18 GPa if it is reinforced; similar to human bone (90-100 MPa or 10-14 GPa), unlike titanium (14). The soft tissue healing around the implant abutment is important (16); the material and surface properties have a major effect on the cellular and bone response (17). Most studies investigate the relationship between the effects of titanium and zirconia abutments. However, there are few articles in the literature that discuss the temporary or permanent use of polymers as abutments (18).

Another factor to be considered is the surface roughness (Ra) of the abutment. This plays an important role in bacterial plaque accumulation and adhesion. Ra greater than 0.2µm tends to favour bacterial adhesion (19). According to D’Ercole *et al*. PEEK has a greater anti-adhesive and/or bactericidal effect against *Streptococcus oralis* than Ti. This action, which lasts 24-48 hours, may play an important role in the prevention of pathologies related to biofilm formation, such as peri-implantitis (20). Hydrophobic abutment surfaces reduce cell adhesion, so a hydrophilic surface is necessary for cell interaction with surrounding tissues (21). Generally, a material is considered hydrophilic when the contact angle of a water droplet with the surface is less than 90° and hydrophobic if it exceeds 90° (22).

It is widely accepted that the geometric structure of the material’s surface can directly proportionally regulate hydrophilicity and roughness, which synergistically affects cellular behaviour and osseointegration (23). Most polymers have a low surface energy, which makes them bionert materials.For this purpose, the surfaces are subjected to an abrasion process with air particles and acid treatment, thus stimulating osteoblastic activity and improving bone-to-implant contact (BIC) (31). They can also be modified with laser, and better fixation of gingival fibroblasts is obtained than on non-laser-modified surfaces, both in PEEK and Ti (24).

According to a study by Najeeb *et al*. (7), bioactive coating substances such as tricalcium phosphate (TCP), titanium dioxide (TiO2), calcium hydroxyapatite (HAp), HAF, aluminium oxide and silk fibroin are also used. It have been shown that the surface bioctivity of PEEK is increased when treated with sulphuric acid (H2SO4); sulphonated porous layers are created (25) This procedure is simple, effective and does not cause great damage to the mechanical properties of the material. The mechanical strength and modulus of elasticity of PEEK abutments can be improved with the use of carbon fibres (CFR-PEEK) (4,20). Qin *et al*. (26) state that graphene oxide promotes osteogenesis, improves hydrophilicity, microroughness and nanostructure. In addition, it is neither cytotoxic nor causes systemic toxicity. They suggest it is a good material for implants and abutments.

The existence of this novel material opens up new possibilities and alternatives for its use in dentistry, for which reason we believe it is appropriate to carry out an updated systematic review on the subject. The aim of this systematic review is to analyse and make a qualitative synthesis of the available literature on the mechanical and functional properties of PEEK as an alternative material to titanium for implant abutments.

## Material and Methods

-Statement and protocol

This systematic review was conducted according to the Preferred Reporting Items for Systematic Reviews and Meta-Analyses (PRISMA) guidelines (27). The protocol of this review was included in the international prospective registry of systematic reviews, PROSPERO (ID 274834). The clinical question set for the search strategy was organised using the PICO question.

-Search terms and strategy

A search of the Medline (PubMed), SciELO, Cochrane and Web of Science (WOS) databases was carried out on 20 February 2021 and the search was updated on 19 March 2021. A complementary search of grey literature was also conducted in Google Scholar so that, if valuable information existed, it could be contributed to the present work.

The following search terms were used: “Polyetheretherketone AND abutment”, “Dental implant abutment AND PEEK”, “Peek Implant abutment”, “Peek abutment AND titanium abutment” and “PEEK implant AND titanium implant”. The Boolean operator AND was used.

-Inclusion and exclusion criteria

A number of inclusion and exclusion criteria were applied for the selection of articles.

Inclusion criteria

- Articles published within the last 5 years.

- Articles related to PEEK material and its use as an abutment.

- In vitro experimental articles.

- Articles written in English.

Exclusion criteria

- Articles that have not been published in the last 5 years.

- Articles that do not have an abstract or summary.

- Systematic reviews, letters to the editor, and clinical cases.

-Eligibility Criteria

The following specific question was formulated according to the principle of Participants, Intervention, Control, Outcome (PICO strategy): “Are the properties of PEEK implant abutments comparable to or better than titanium?

- P: prosthetic implant rehabilitation

- I: PEEK implant abutment

- C: titanium implant abutment

- O: Fracture resistance rate

-Study selection process

The results obtained from the search, using the terms mentioned above, were compiled in BibTex and NBIB format for subsequent export to Mendeley Desktop 1.19.8; a bibliographic manager that has been used in order to discard duplicate articles.

Articles were chosen by reading only the title and abstract of the study. If they were not of interest and did not correspond to the inclusion criteria mentioned above, they were discarded. If the abstract did not provide enough information to make a decision to include or exclude the article, it was read in full.

Finally, the full text of the articles that met the above-mentioned eligibility requirements was read.

-Data extraction

For the bibliometric analysis, the following variables were recorded for each article: author and year of publication. The variables recorded for the study methodology were the following: sample description (implant type, materials and number), abutment and crown placement site, bone type, cortical thickness, maximum applied force and dynamic fatigue. And the variables recorded for the results were torque loss, fracture load and mean surface roughness value.

-Quality analysis

The studies included in the present systematic review were independently assessed for risk of bias using the modified CONSORT guide (28) for reporting *in vitro* studies of dental materials, noting which parameters were met and which were not. In addition, the percentage of compliance with the items for each of the studies was calculated.

## Results

-Search and selection of articles

**Figure 1:**
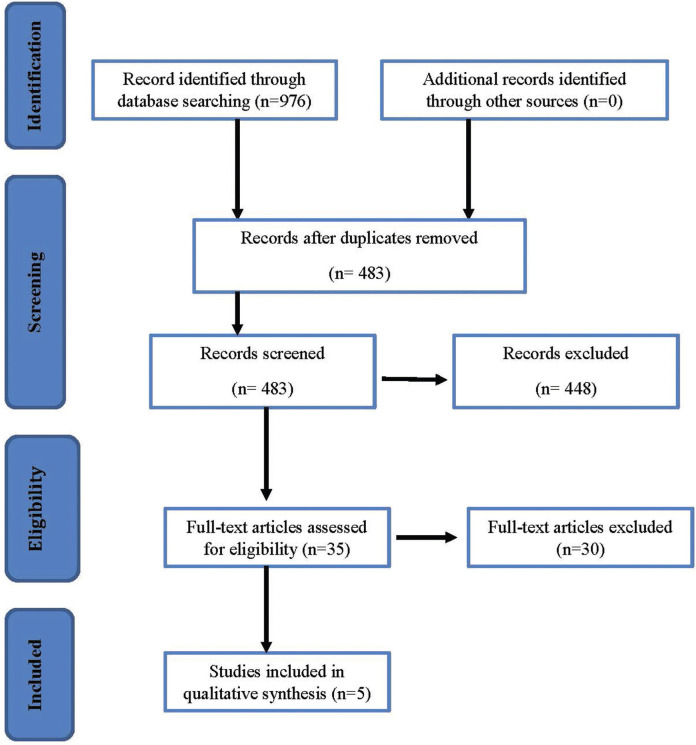
Flowchart of the serach conducted in this sytematic revew. Based on the Preferred Reporting Items for Systemic Reviews and Meta-Analyses (PRISMA) flowchart (27).

The number of references obtained from the databases is 976; 421 from MedLine, 68 from Cochrane and 487 from Web of Science (identification phase). The search in Google Scholar and SciELO yielded no results and no additional sources were consulted. The number of studies was not increased after the update search. In the screening phase, duplicates (493 articles) were discarded manually in the reference management software, reducing the number of articles to 483. After reading the titles and abstracts,448 references were excluded. Thirty-five full-text articles were analysed, of which five were included in this qualitative synthesis (Fig. 1).

-Evaluation of the mechanical and functional properties of PEEK and Titanium abutments.

Table 1 summarises the methodology of the articles selected for this systematic review.

**Table 1:**
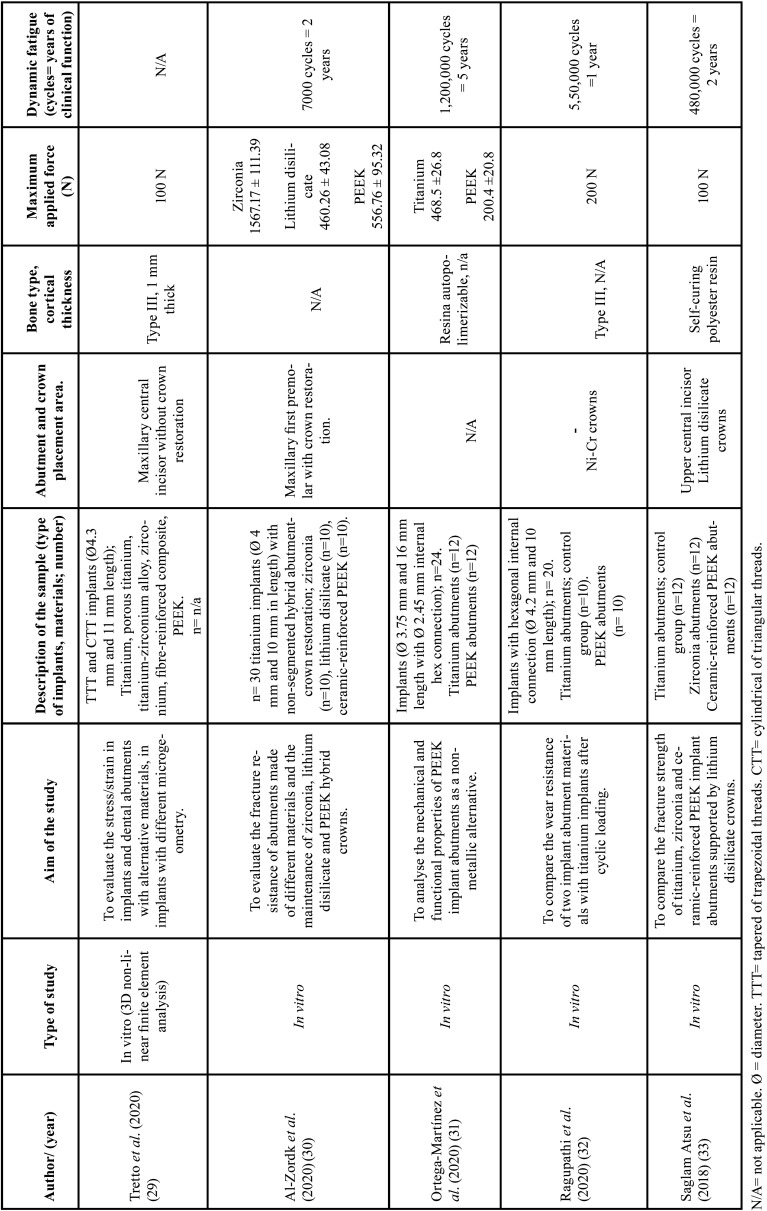
Summary of the methodology of the included studies.

Wentz Tretto *et al*., (29) conducted a study whose objective was to evaluate the stress or deformation of implants and dental abutments with different materials using non-linear finite element analysis (FEA). They used two types of implants; tapered trapezoidal threaded (TTT) and cylindrical triangular threaded (CTT), and 6 different materials. The rehabilitation of a maxillary central incisor with a single crown on a customised abutment was simulated and a force of 100 N, perpendicular to the long axis of the implant, was applied to the type III bone (according to the Misch classification). Three different possibilities were analysed:

(a) Titanium prosthetic abutment combined with implants of different materials (titanium, porous titanium, titanium-zirconium, zirconium, RFC and PEEK).

b) Implants and prosthetic abutments of the same material in one piece (titanium, zirconia, RFC and PEEK).

c) Titanium implant combined with different prosthetic abutment materials (titanium, zirconia, RFC and PEEK).

Using computed tomography (CT) images, the maxillary bone was established at a slice thickness of 1 mm; only the anterior portion of the maxillary bone structure was used.

The stress in the peri-implant bone tissue was inversely proportional to the elastic modulus of the implant material, independent of implant macrogeometry. There is more stress in the PEEK and RFC implant when titanium abutments are used.

However, less stress is observed in PEEK and RFC structures in prosthetic abutments when they are used as one piece.

As for the titanium implant with a combination of different materials as prosthetic abutments, PEEK and RFC transmit higher stress concentration to the implant and peri-implant bone tissue (both macrogeometries, although in the CTT configuration there is less stress).

Al-Zordk *et al*., (30) performed an *in vitro* study with 30 titanium implants with non-segmented abutment-crown hybrid restorations of zirconia, lithium disilicate and ceramic-reinforced PEEK; 10 crowns of each material.

Thirty epoxy models simulating the jaw were made. A surgical guide was designed with a 3D printer so that the extraction process of the first premolar could be carried out in the same way. A titanium implant with its corresponding base was attached to each model. Everything was sprayed together with an anti-reflective powder. The restorations were milled from blocks using CAD-CAM and cemented with resin cement. A torque of 25 Ncm was applied.

All samples were subjected to an artificial thermal ageing process of 7000 cycles corresponding to 2 years of clinical function. After this process, vertical forces were applied to each specimen until breakage.

The following variables were analysed; torque, loosening, torque loss and percentage torque loss of all types of crown-abutment restorations.

The restorations with the highest mean torque loss value were zirconia (2.70 ± 0.59 Ncm) with a loosening loss of 22.38 ± 0.68 Ncm and those with the lowest mean loss value were PEEK (2.55 ± 0.50 Ncm) with a loosening loss of 22.61 ± 0.59 Ncm. There was no significant difference between the different groups in terms of torque loosening (*p*=0.68), torque loss (*p*=0.80) and percentage loss of touch (*p*=0.79).

The restorations showing the highest mean value of maximum fracture load were zirconia (1567.17 ± 111.39 N), followed by PEEK (556.76 ± 95.32 N), leaving lithium disilicate (460.26 ± 43.08 N) in last position. According to the Posthoc Tukey test, there is a significant difference between zirconia and PEEK (*p* < 0.001) and between lithium disilicate and zirconia crown-abutment restorations. However, there is no significant difference between lithium disilicate and PEEK (*p* = 0.05).

Ortega-Martinez *et al*., (31) evaluated 2 different implant abutments; PEEK and titanium grade 5 (control group). These were attached to hex-connected titanium implants and retightened after 10 minutes. To simulate oral conditions, the implants were pre-impregnated in polymer resin and placed perpendicularly 3.0 ±0.1 mm above bone level in a self-curing resin with a modulus of elasticity greater than 3 GPa (7.28 ±0.89 GPa); recommended according to ISO 14801:2016. The dynamic fatigue test was then carried out at a frequency of 15 Hz for 1.2 million cycles (equivalent to approximately 5 years of occlusal function) at room temperature and under dry conditions.

The maximum applied load was calculated according to ISO 14801:2016; reducing until the PEEK specimens reached the expected service life. Both PEEK and titanium abutments were evaluated under the same conditions.

After uniaxial compression testing, the mechanical performance of the titanium abutments was found to be significantly higher than that of the PEEK abutments in terms of peak strength and fracture displacement. PEEK abutments showed 56% less peak force and 25% less fracture displacement than titanium abutments. However, the plastic deformation was concentrated at the base of the abutment, unlike titanium; it was concentrated at the internal connection of the implant.

In the dynamic fatigue test, no titanium or PEEK specimens fractured.

The abutments were soaked in a methylene blue solution to check for torque loss and microleakage. There was a torque loss of approximately 10% for titanium abutments, rising to 50% for PEEK abutments.

Regarding microleakage, titanium abutments had lower values with or without dynamic fatigue; except for 2 specimens subjected to dynamic fatigue which showed traces of staining in the shoulder area of the connection. Regarding the PEEK abutments, 7 specimens showed staining at the connection shoulder level without dynamic fatigue and 5 specimens without staining. All PEEK abutments with dynamic fatigue showed microleakage at the shoulder or hexagonal connection.

Another study by Ragupathi *et al*., (32) compared the wear resistance of two titanium implant abutment materials with titanium implants after cyclic loading. Twenty internally connected titanium implants were used. They were inserted into a block of self-curing acrylic resin, leaving 2 mm of implant shoulder above the resin to mimic minimal type III cancellous bone loss. Titanium (Group I) and PEEK (Group II) implant abutments were loaded with a torque of 35 Ncm.

All samples were individually 3D scanned prior to cyclic loading to obtain an average surface roughness value and examined by scanning electron microscopy (SEM) using different magnifications depending on the area of interest (x30, x200, x500, x1000). In addition to the use of energy dispersive X-ray spectrophotometry (EDS).

After placement and cementation of the Ni-Cr crowns on both types of abutments, cyclic loading was performed in a dry environment. A sine wave at 2 Hz was applied for a load of up to 200 N simulating human masticatory frequency and loads. The loading angle represents a class I occlusion. The cycle time was 72 hours (with a 2-hour pause every 21 hours), 550,000 cycles were simulated, equivalent to 1 year of function.

The abutments were then separated from their implants to visually inspect for deformation and damage. They were then examined with SEM, 3D surface profilometry and EDS. The surface roughness (Ra) values before and after loading of Group I (-0.073 μm) were lower than Group II samples (-0.0004 μm); it is statistically insignificant (*p*= 0.272).

SEM micrographs of Group I taken before cyclic loading show gaps indicating a rough surface and after cyclic loading micro-irregularities and decreased gaps were visible, indicating a higher rate of wear. In Group II before cyclic loading, sparsely distributed microgrooved irregularities with a smooth surface were observed. After cyclic loading, sparsely distributed striations appeared. So there is more wear than the preload.

The EDS results of the titanium abutment before loading indicate the presence of 100% titanium and after this procedure it was reduced to 83.94%. For the PEEK abutment, 100% carbon was observed before loading and 66.04% after loading. Generally, a higher wear resistance is shown for titanium abutments compared to PEEK abutments, but there is not a large statistical difference.

Saglam Atsu *et al*., (33) conducted a study aimed at comparing the fracture strength of titanium, zirconia and ceramic-reinforced PEEK (RPEEK) implant abutments supported by lithium disilicate crowns.

Thirty-six implant abutments were divided as follows:

(a) 12 titanium abutment specimens (control group).

b) 12 specimens of zirconia abutments.

c) 12 specimens of ceramic-reinforced PEEK abutments.

All abutments used in this study have the same dimensions; a diameter of 3.5 mm and a length of 9 mm with an internal hex connection of 2.2 mm in length. All abutments were screw-retained. Analogue implants (4 mm diameter and 14 mm length) were embedded in a self-curing polyester resin with an elastic modulus of 2.6 GPa (to mimic an elastic reaction of the surrounding bone during loading). To simulate occlusal loading they were positioned at an angulation of 30° and joined to the abutments by applying a torque of 25 Ncm, the same torque was re-applied after 10 minutes to compensate for the loss of preload.

The 36 lithium disilicate crowns have a standard size for all abutments; a height of 11 mm and a width of 8.5 mm and were cemented with resin. A 6 mm diameter stainless steel ball was used as an antagonist. To mimic oral conditions, they were immersed in distilled water at room temperature throughout the test.

A force of 100 N and a frequency of 1.6 Hz were applied to all samples to simulate the chewing of two clinical years. A stereoscope was used to check for the presence or absence of fractures. The samples without fractures were subjected to a dynamic fatigue of 2,000 cycles and re-examined.

The results reveal that the group with the highest fracture resistance is Ti (782.80+- 120.9 N), followed by Zr (623.93+-97.4 N) and finally RPEEK (602.93+-121 N) (Table 2).

**Table 2:**
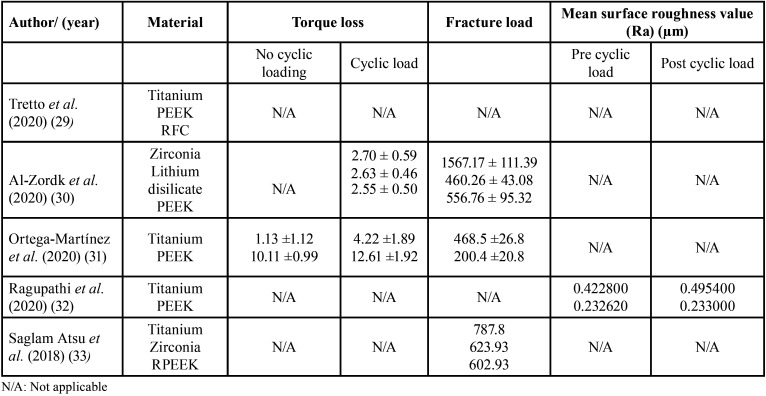
Summary of results of included studies.

-Quality analysis

The results of the quality analysis obtained using the modified CONSORT guide (28) mentioned above are given in Table 3. The mean compliance of the items of the included studies was 69.6%, with a maximum of 78% and a minimum of 64%. Items 6-9 (related to the randomisation method) are not reported in any article. However, items 1-3,5,13,14 (study structure, sample determination, funding sources and availability of the full protocol) appear in all included articles.

**Table 3:**
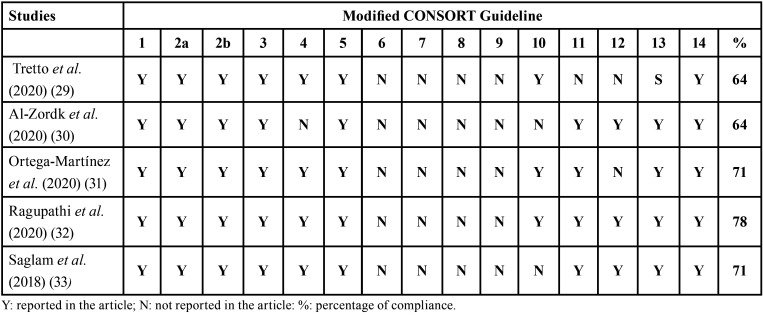
Results of the assessment of *in vitro* studies using the modified CONSORT checklist (28).

## Discussion

PEEK is considered as an alternative to titanium and ceramics (4). Because it offers numerous desirable characteristics such as aesthetics (whitish tooth-like colour), biocompatibility, fracture resistance, high mechanical properties and chemical stability, and anti-adhesive and/or anti-bacterial effect (3,12,20).

The ideal characteristics of a definitive or provisional material are: high fracture resistance, resistance to tensile forces, distribution of chewing forces to the surrounding peri-implant tissues, and no loosening of screws or microcracks. To improve the biomechanics of PEEK, some studies reinforce them with carbon fibre (CFR/PEEK) providing a stronger temporary or final implant abutment. However, CFR/PEEK has clinically relevant disadvantages, such as its dark colour and soft tissue swelling when the carbon fibres are detached from the PEEK core (13).

Despite limited information on ceramic-reinforced PEEK (RPEEK), there are articles using it as an implant abutment, as it has good biomechanical characteristics and is biocompatible. In a case report by Al-Rabab’ah *et al*. this material was used as a permanent abutment. After two years of follow-up, the bone and soft tissue around the implant remained stable (34).

In the article by Zordk *et al*. (30) it is mentioned that one of the important concerns in implant-supported restorations is the fixation technique; it can be done by means of a retaining screw or cement-screws. The last one has better passive compliance, aesthetics and load distribution. Furthermore, Zarone *et al*. (35) state that the load-bearing capacity of the cement-screw system is higher than that of the screw-retained system. Freitas *et al*. (36) report that cement-retained restorations with cemented internal connections have higher fracture resistance, which is why cemented abutments with internal connection were used in the article by Atsu *et al*. However, this technique has the risk that excess cement that persists in the submucosal region may develop peri-implant disease (mucositis or peri-implantitis). The prevalence of this condition ranges from 1.9 to 75% of cemented implant-supported restorations (30).

Another variable to be considered is the physiological bite force. Maximum occlusal forces of 150 to 300 N have been recorded for incisors (37), between 200-445 N for premolars and up to 900 N for molars (38).

Fracture resistance is higher in abutments attached to crowns than in those where the crown and abutment are separated. (30) In the study by Atsu *et al*. (33), the fracture toughness of Ti (943.67 N) is higher than that of Zr and RPEEK (770.1 N). However, there is no significant difference between the latter two (*p*=0.001). In the article by Ortega *et al*., (31) the mean fracture toughness takes values of 468.5 N for the Ti abutment and 200.4 N for the PEEK abutment. Fatigue tests were performed at 140 N with 1.2 million cycles, equivalent to 5 years of function, and there was no failure of these abutments. However, when the load was increased to 160 N, the PEEK abutments did not exceed 89,338 cycles (equivalent to 4-5 months of occlusal function).

It has been observed that PEEK acts as a “sacrificial material”; it absorbs all plastic deformation by breaking before the implant or internal screw. Whereas, in the case of titanium abutments, the plastic deformation is concentrated in the internal connection, thus compromising the viability of the implant after overloading (31).

In the study by Zordk *et al*. (30) there were significant differences between screw tightening and loosening within each group. There is a possibility that thermal ageing may have impaired the integrity of the resin cement in load transfer. The same occurrence occurs in the study by Ortega *et al*., (31); a large vertical displacement and plastic deformation was shown during the dynamic fatigue test, which resulted in loss of torque. The increase in material temperature also favours this loss.

Lack of fit at the implant-abutment interface or loss of torque is related to: micromovement during mastication, mismatch between implant and abutment, and incorrect torque application. (39) This leads to microinfiltration or microleakage. The presence of this phenomenon allows bacterial colonisation that can trigger a peri-implant inflammatory process. (40) According to the study by Ortega *et al*. (31), the incidence of microleakage is lower in titanium abutments than in PEEK abutments. This is probably because there is a higher incidence of screw loosening and plastic deformation in the latter. Plastic deformation is known to cause premature screw loosening and microleakage.

Of the studies selected for the present work, only the study by Ragupathi *et al*., (32) compares the surface roughness values (Ra value) between titanium (group I) and PEEK (group II) after cyclic loading. The difference between the surface roughness value before and after cyclic loading was calculated to evaluate the wear rate. Using the independent t-test, it was observed that the mean difference of surface roughness before and after cyclic loading of group I (-0.073 μm) is lower than the samples of group II (-0.0004 μm); it is statistically insignificant (*P*=0.272).

-Limitations

Admittedly, the present systematic review contains only 5 *in vitro* articles, but it should not be forgotten that this is a topic that is only recently being studied and there is a very limited literature on this material. Moreover, the methodological heterogeneity of these articles makes it difficult to reach a clear conclusion.

Further studies are desirable to better understand the long-term behaviour of the material under both *in vivo* and *in vitro* intraoral conditions by increasing the sample size. It is advisable to open lines of research to study dynamic fatigue, fracture resistance, torque loss and bacterial adhesion in a humid environment that simulates the intraoral situation, to use highly sensitive spectrometric techniques to determine microleakage with dyes and to know the behaviour of PEEK when opposed to different restorative materials as antagonists. It would also be interesting to investigate surface treatment techniques that improve the bioactivity of PEEK before using it as an abutment.

## Conclusions

PEEK implant abutments do not have sufficient biomechanical requirements to replace the definitive titanium abutment, however, it is considered as an alternative material. PEEK abutments can be used as temporary abutments, especially in the anterior region (where lower masticatory forces exist) and in patients without parafunction.

## References

[1] Brånemark P, Hansson B, Adell R, Breine U, Lindström J, Hallén O (1977). Osseointegrated implants in the treatment of the edentulous jaw. Experience from a 10-year period. Scand J Plast Reconstr Surg Hand Surg.

[2] Ma R, Tang T (2014). Current strategies to improve the bioactivity of PEEK. Int J Mol Sci.

[3] Wiesli MG, Ozcan M (2015). High-Performance Polymers and Their Potential Application as Medical and Oral Implant Materials: A Review. Implant Dent.

[4] Andreiotelli M, Wenz HJ, Kohal RJ (2009). Are ceramic implants a viable alternative to titanium implants? A systematic literature review. Clin Oral Implants Res.

[5] Papathanasiou I, Polyzois G (2016). The use of a modified polyether-ether-ketone (PEEK) as an alternative framework material for removable dental prostheses. A clinical report. J Prosthodont.

[6] Stawarczyk B, Beuer F, Wimmer T, Jahn D, Sener B, Roos M (2013). Polyetheretherketoneda suitable material for fixed dental prostheses?. J Biomed Mater Res B Appl Biomater.

[7] Najeeb S, Zafar MS, Khurshid Z, Siddiqui F (2016). Applications of polyetheretherketone (PEEK) in oral implantology and prosthodontics. J Prosthodont Res.

[8] Silthamitag P, Chaijareenont P, Tattakorn K, Banjongprasert C, Takahashi H, Arksornnukit M (2016). Effect of surface pretreatments on resin composite bonding to PEEK. Dent Mater J.

[9] Altmeyer J, Dos Santos JF, Amancio-Filho ST (2014). Effect of the friction riveting process parameters on the joint formation and performance of Ti alloy/short-fibre reinforced polyether ether ketone joints. Mater Design.

[10] Green S, Schlegel J (2001). A polyaryletherketone biomaterial for use in medical implant applications. Polym for the Med Ind Pro.

[11] Kurtz SM, Devine JN (2007). PEEK biomaterials in trauma, orthopedic, and spinal implants. Biomaterials.

[12] Schwitalla AD, Spintig T, Kallage I, Müller W (2015). Flexural behavior of PEEK materials for dental application. Dent Mater.

[13] Schwitalla AD, Zimmermann T, Spintig T, Kallage I, Müller W (2017). Fatigue limits of different PEEK materials for dental implants. J Mech Behav Biomed Mater.

[14] Panayotov IV, Orti V, Cuisinier F, Yachouh J (2016). Polyetheretherketone (PEEK) for medical applications. J Mater Sci Mater Med.

[15] Lee W, Koak J, Lim Y, Kim S, Kwon H, Kim M (2012). Stress shielding and fatigue limits of polyetheretherketone dental implants. J Biomed Mater Res B Appl Biomater.

[16] Rompen E, Domken O, Degidi M, Pontes AE, Piattelli A (2006). The effect of material characteristics, of surface topography and of implant components and connections on sof t tissue integration: A literature review. Clin Oral Implants Res.

[17] Canullo L, Annunziata M, Pesce P, Tommasato G, Nastri L, Guida L (2021). Influence of abutment material and modifications on peri-implant soft-tissue attachment: A systematic review and meta-analysis of histological animal studies. J Prosthet Dent.

[18] Alp G, Johnston WM, Yilmaz B (2019). Optical properties and surface roughness of prepolymerized poly (methyl methacrylate) denture base materials. J Prosthet Dent.

[19] D'Ercole S, Cellini L, Pilato S, Di Lodovico S, Iezzi G, Piattelli A (2020). Material characterization and Streptococcus oralis adhesion on Polyetheretherketone (PEEK) and titanium surfaces used in implantology. J Mater Sci Mater Med.

[20] Gittens RA, Scheideler L, Rupp F, Hyzy SL, Geis-Gerstorfer J, Schwartz Z (2014). A review on the wettability of dental implant surfaces II: Biological and clinical aspects. Acta Biomater.

[21] Sartoretto SC, Alves ATNN, Resende RFB, Calasans-Maia J, Granjeiro JM, Calasans-Maia MD (2015). Early osseointegration driven by the surface chemistry and wettability of dental implants. J. Appl Oral Sci.

[22] Gittens RA, Olivares-Navarrete R, Schwartz Z, Boyan BD (2014). Implant osseointegration and the role of microroughness and nanostructures: lessons for spine implants. Acta Biomater.

[23] Kurtz SM, Devine JN (2007). PEEK biomaterials in trauma, orthopedic, and spinal implants. Biomaterials.

[24] Gheisarifar M, Thompson GA, Drago C, Tabatabaei F, Rasoulianboroujeni M (2021). In vitro study of surface alterations to polyetheretherketone and titanium and their effect upon human gingival fibroblasts. J Prosthet Dent.

[25] Yuan B, Cheng QW, Zhao R, Zhu XD, Yang X, Yang X (2018). Comparison of osteointegration property between PEKK and PEEK: effects of surface structure and chemistry. Biomaterials.

[26] Qin W, Li Y, Ma J, Liang Q, Cui X, Jia H (2020). Osseointegration and biosafety of graphene oxide wrapped porous CF/PEEK composites as implantable materials: The role of surface structure and chemistry. Dent Mater.

[27] Moher D, Liberati A, Tetzlaff J, Altman DG (2009). Preferred reporting items for systematic reviews and meta-analyses: the PRISMA statement. Ann Intern Med.

[28] Faggion CM Jr (2012). Guidelines for reporting pre-clinical in vitro studies on dental materials. J Evid Based Dent Pract.

[29] Tretto PHW, Dos Santos MBF, Spazzin AO, Pereira GKR, Bacchi A (2020). Assessment of stress/strain in dental implants and abutments of alternative materials compared to conventional titanium alloy-3D non-linear finite element analysis. Comput Methods Biomech Biomed Engin.

[30] Al-Zordk W, Elmisery A, Ghazy M (2020). Hybrid-abutment-restoration: effect of material type on torque maintenance and fracture resistance after thermal aging. Int J Implant Dent.

[31] Ortega-Martínez J, Delgado LM, Ortiz-Hernández M, Punset M, Cano-Batalla J, Cayon MR (2022). In vitro assessment of PEEK and titanium implant abutments: Screw loosening and microleakage evaluations under dynamic mechanical testing. J Prosthet Dent.

[32] Ragupathi M, Mahadevan V, Azhagarasan NS, Ramakrishnan H, Jayakrishnakumar S (2020). Comparative evaluation of the wear resistance of two different implant abutment materials after cyclic loading - An in vitro study. Contemp Clin Dent.

[33] Atsü SS, Aksan ME, Bulut AC (2019). Fracture Resistance of Titanium, Zirconia, and CeramicReinforced Polyetheretherketone Implant Abutments Supporting CAD/CAM Monolithic Lithium Disilicate Ceramic Crowns After Aging. Int J Oral Maxillofac Implants.

[34] Al-Rabab'ah M, Hamadneh W, Alsalem I, Khraisat A, Abu Karaky A (2019). Use of High-Performance Polymers as Dental Implant Abutment and Frameworks: A Case Series Report. J Prosthodont.

[35] Zarone F, Sorrentino R, Traini T, Di Lorio D, Caputi S (2007). Fracture resistance of implant-supported screw-versus cement-retained porcelain fused to metal single crowns: SEM fractographic analysis. Dent Mater.

[36] Freitas ACJ, Bonfante EA, Rocha EP, Silva NRF, Marotta LPC (2011). Effect of implant connection and restoration design (screwed vs. cemented) in reliability and failure modes of anterior crowns. Eur J Oral Sci.

[37] Morneburg TR, Proschel PA (2002). Measurement of masticatory forces and implant loads: a methodologic clinical study. Int J. Proshodont.

[38] Li Xu, Zhu Z, Li Z, Zhou J, Chen W (2019). All-ceramic premolar guiding late retains resin-bonded fixed partial dentures. J Stomatol.

[39] Wachtel A, Zimmermann T, Spintig T, Beuer F, Müller W, Schwitalla AD (2016). A novel approach to prove bacterial leakage of implant-abutment connections in vitro. J Oral Implantol.

[40] Park J, Baek C, Heo S, Kim S, Koak J, Kim S (2017). An in vitro evaluation of the loosening of different interchangeable abutments in internal-connection type implants. Int J Oral Maxillofac Implants.

